# Psychometric Assessment of the Metamorphopsia Questionnaire in Patients with Macular Diseases Receiving Anti-Vascular Endothelial Growth Factor Treatment

**DOI:** 10.3390/jcm15082867

**Published:** 2026-04-09

**Authors:** Francis W. B. Sanders, Jennifer H. Acton, Barbara Ryan, Colm McAlinden

**Affiliations:** 1 Department of Ophthalmology, Prince Philip Hospital, Hywel Dda University Health Board, Llanelli SA14 8QF, UK; 2 School of Optometry and Vision Sciences, Cardiff University, Cardiff CF24 4HQ, UK; 3 Department of Ophthalmology, Princess of Wales Hospital, Bridgend CF31 1RQ, UK; 4 Wenzhou Medical University, Wenzhou 325015, China; 5 Eye and Ear Nose & Throat Hospital, Fudan University, Shanghai 200437, China

**Keywords:** AMD, PROM, Rasch, Diabetic Retinopathy

## Abstract

**Background:** The metamorphopsia questionnaire (MeMoQ) is an established patient-reported outcome measure (PROM) in the context of macular disease. However, its performance has not been proved in those being treated for various macular conditions with intravitreal anti-vascular endothelial growth factor (Anti-VEGF). The objective was to eliminate misfitting items, enhance measurement precision, and ensure optimal response categorisation. **Methods:** Rasch analysis was performed iteratively on 2286 responses from patients with macular diseases being treated with Anti-VEGF to optimise the MeMoQ. Fit statistics, reliability indices, person and item separation measures, and principal component analysis (PCA) of residuals were assessed to determine the optimal model. This study was conducted in an outpatient clinic specialising in retinal diseases in Hywel Dda University Health Board. **Results:** Misfitting items were removed in successive iterations, leading to optimised category probability curves and stable fit statistics for the MeMoQ. The resulting model for all responses included two final items, with person separation remaining inadequate reducing from 1.23 to 1.12 and reliability from 0.60 to 0.56. Category probability curves demonstrated good ordering of response variables with Andrich thresholds separated by >1.2 logits. In the subgroups of neovascular age-related macular degeneration and diabetic macular oedema person separation remained below two and reliability remained low. **Conclusions:** Rasch analysis demonstrated that the MeMoQ was not a valid or reliable PROM in this patient population. Therefore, the MeMoQ may not provide a reliable index of patient’s perception and visual experience when undergoing Anti-VEGF treatment.

## 1. Introduction

Diseases affecting the macula are traditionally associated with symptoms of metamorphopsia, a subjective phenomenon which is difficult to quantify with an objective metric in the busy clinic setting [1]. This, however, does not detract from the significant effects it has on patients in terms of their subjective quality of vision and perceived impact on their daily life.

Patient-reported outcome measures (PROMs) provide a structured assessment of the multifaceted effects of specific diseases from the perspective of the individual [2,3,4,5]. This information allows for improved patient-focused care and treatment selection based on their particular issues [2,5]. This is particularly true for PROMs which have been designed and validated to operate robustly and reliably in a particular population of patients. This is of clinical significance in the context of patients suffering from metamorphopsia, as even with mild degrees of vision loss, the symptoms associated with metamorphopsia, such as distortion of faces, can significantly impact on social interactions and confidence [6]. Although a number of PROMS have been developed for those undergoing anti-vascular endothelial growth factor (Anti-VEGF) therapy [7,8,9,10,11,12], none specifically address metamorphopsia. This highlights the need for addressing such symptoms in a robust and reliable PROM to validate patients’ symptoms and justify surgical or therapeutic intervention and their associated risks.

The metamorphopsia questionnaire (MeMoQ) is a PROM designed to assess subjective metamorphopsia in the context of macular disease [13]. The questionnaire was developed in Japan and hence was validated in Japanese originally. Subsequently it was translated into English, and questions that would not be applicable outside of individuals familiar with traditional Japanese architecture were eliminated [14]. This has led to its use in multiple studies of macular disease in which the patient’s experience of metamorphopsia is key to the outcomes required of the disease process, surgical intervention or pharmacological therapy [13,14,15].

The English translation of the MeMoQ has not been validated in the population of patients undergoing intravitreal Anti-VEGF therapy. With this in mind, the therapeutic effects of Anti-VEGF effectively leading to reduced intraretinal and subretinal fluid ameliorate symptoms of metamorphopsia to varying degrees, although some studies in nAMD do not show a correlation between objectively measured metamorphopsia and central retinal thickness [16,17,18,19,20]. This raises questions about its psychometric performance within this population, as well as the reliability of the questionnaire across a spectrum of conditions from neovascular age-related macular degeneration (nAMD) to diabetic macular oedema (DMO), to retinal vein occlusion (RVO) complicated by cystoid macular oedema (CMO). This will be of growing concern in the English-speaking world where reliable PROMs will be vital to the delivery of cost-effective healthcare to an ageing population, with the associated rising incidence of nAMD, RVO and DMO [2,4,5,21,22].

As a result the current study aimed to investigate the psychometric properties of the MeMoQ within a heterogenous population of patients being treated with Anti-VEGF for a range of conditions including nAMD, RVO and DMO.

## 2. Materials and Methods

### 2.1. The Questionnaire

The MeMoQ is an 8-item questionnaire adapted from the 10-part 2011 questionnaire [13,14] with the prior misfitting item removed (Q8: “*Do people’s faces appear distorted to you? Do the parts of the faces also appear missing?”*) and removal of one question not relevant to the European population under investigation (Q4 “*Do the columns in your Japanese style rooms appear distorted or tilted to you?*”). This is summarised in Table 1. All items had standardised responses (“not at all,” 0 points; “a little,” 1 point; “moderately,” 2 points; and “a great deal,” 3 points). As per the original publication if the question was not deemed relevant to the individual they would select “none of the above” and no score would be attributed to that question [13].

### 2.2. Population

All participants were individuals under the care of the Hywel Dda University Health Board (HDUHB), Wales, UK, receiving intravitreal anti-vascular endothelial growth factor (Anti-VEGF) therapy for various macular conditions. Automated electronic forms were sent to patients using an established PROM delivery platform (DrDoctor Ltd., London, UK). Participants completed the questionnaires electronically which were automatically collated. Participants completed the questionnaire once every 3 months. A total of 2286 responses were collated between 1 November 2020 and 1 April 2024 from a total of 1164 patients who responded on average 1.96 times, ranging from 1 to a maximum of 13 responses.

Subgroups of patients were identified based on their underlying clinical diagnosis with 1077 responses from patients with nAMD, 281 responses from patients being treated for DMO, 282 responses from patients being treated for macular oedema associated with RVO and 646 with a range of other macular pathologies for which they were receiving Anti-VEGF.

### 2.3. Ethical Review

The study was approved by the Research and Development Department of HDUHB and the need for further review by a national Research Ethics Committee was waived. The study was conducted in accordance with the tenets of the Declaration of the Helsinki with permission provided by patients for the use of questionnaire responses for service and questionnaire evaluation.

### 2.4. Statistical Analysis

Rasch analysis was used to assess the psychometric properties of the MeMoQ within both the pooled responses of the entire population as well as subgroup analysis of each of the groups of nAMD, DMO and RVO, with each response being analysed as an independent entity. Rasch modelling assesses the difference between item difficulty and a person’s ability in a probabilistic manner, for more information on the details we refer readers to this review paper [23,24]. Briefly, the difference between the item difficulty and the patient’s ability represents the functional reserve of the item measure in the questionnaire. The probability of “success” or “failure” at an item is converted from the raw scores, with the natural logarithm of this ratio measuring the difference between person ability and item difficulty [25]. This generates a common unit of measurement—the logit—for item difficulty as well as person ability. In the current analysis we used the Andrich rating scale model to assess the MeMoQ using Winsteps Rasch analysis software (Version 3.93.2, Winsteps, Chicago, IL, USA).

The MeMoQ was assessed for response category ordering, item fit statistics, principal component analysis (PCA) of residuals and person separation index as outlined below.

1. The response category ordering was visually inspected via category probability curves for appropriate ordering of responses for all items in each questionnaire. Any disorderly responses would have been collapsed to improve category ordering. Additionally, to ensure reliability of ordering a minimum difference of 1.20 logits between Andrich thresholds was utilised [26].
2. Item fit was assessed in terms of infit and outfit characteristics of each item response to check the degree to which items contribute to variance in the Rasch model. Items with a fit statistic means square residual value of 1.0 would indicate expected variance. Thus, for the purposes of the current study those with fit statistics outside the range of 0.80 to 1.20 were sequentially removed to optimise item fit and contribution of each item to measuring the underlying trait being examined. Items with a value of <0.80 represent those with less than 80% of the expected variance and thus contribute less to the model’s functioning and may demonstrate an element of redundancy in items within the questionnaire. On the other hand, those items with mean square residual of >1.20 demonstrate 20% more variance than expected and thus represent items that are potentially measuring an alternate construct than that being examined by the questionnaire. Whilst the clinical relevance of each item was also considered by the research team during refinement and item removal, item fit parameters were used as the main tool for refinement to ensure psychometric integrity.
3. PCA of residual values highlights items that may load onto an alternate construct and as such measure an alternate measurement to that targeted by the questionnaire. This would represent a degree of multidimensionality. If the eigenvalue for the alternative component was greater than 2.0 this represented that at least two items were behaving in such a manner as to measure another characteristic [27]. To identify those items that were contributing to this alternate construct, a threshold of minimum loading of 0.4 was used.
4. Person separation index of 2.0 was used as the minimum standard of discrimination of the questionnaire being assessed. A person separation index measures the discriminative ability of the question to identify differing distinct groups of individuals by ability. For example, a person separation index of two will identify at least three different strata of ability within the population tested.

## 3. Results

Overall, 2286 responses to the MeMoQ were gathered from individuals being treated with intravitreal Anti-VEGF between 1 November 2020 and 1 April 2024.

### 3.1. Entire Population

**Figure 1:**
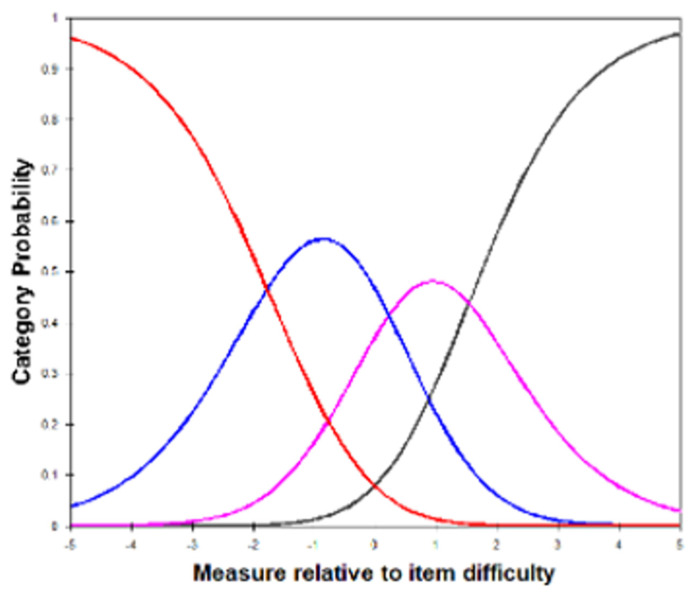
Category probability curve for responses to the metamorphopsia questionnaire (MeMoQ) in the entire population on initial Rasch analysis. Each curve represents the probability of selection of a response category relative to the item’s difficulty. Category 1 (red) is more likely for “easier” items; then, category 2 (blue) is more likely to be selected for “moderately easy”, category 3 (pink) for “moderately difficult” and category 4 (black) for “difficult” items.

Initial Rasch analysis demonstrated good ordering of categories according to the category probability curve with Andrich thresholds spaced by greater than 1.2 logits demonstrating adequate category separation (Figure 1). Person separation was, however, poor, achieving a value of 1.23 for the full eight-item questionnaire. The item-person map provides evidence for mistargeting of the MeMoQ with a differential of 3.27 logits between the means.

Fit statistics for 2286 responses demonstrated a wide range from 0.55 to 2.19 for infit statistics and 0.54 to 2.4 for outfit statistics (Table 2). This suggests that the initial model demonstrates both some redundancy in responses as well as a degree of noise within the responses in terms of increased variance. The model suggested multidimensionality in the full eight-item form with three items demonstrating a loading of >0.4 on the first contrast in the PCA with an eigenvalue of 2.14. All these items describe the patient’s visual experience within the home environment (3. Do the curtain rails in your house appear distorted or tilted to you?; 4. Do the frames of windows or bookshelves appear distorted to you?; 5. Do the lines of the tiles on your bathroom wall appear distorted to you?).

In order to improve the functionality and psychometric properties of the MeMoQ, iterative refinement was performed with elimination of misfitting items in the first instance. For the 2286 responses in this population, only a single refinement was possible using the thresholds of 0.8 to 1.2 logits for the infit statistics, resulting in a two-item questionnaire (1. Do the lines of a crosswalk or the steps of an overpass appear distorted to you?; 2. Do telephone poles or trees appear tilted to you?; Table S1; Figure S1). The resulting model functioned less well than the initial eight-item questionnaire with a person separation of 1.12 and reliability of 0.56. The category probability curves did demonstrate good ordering and separation of items with thresholds spaced by more than 1.2 logits. Item fit did achieve the target of 0.8 to 1.2 logits with infit statistics of 0.93 and 1.03 and outfit of 0.90 and 1.05.

### 3.2. Subgroup Analysis of Patients with nAMD Being Treated with Anti-VEGF

**Figure 2:**
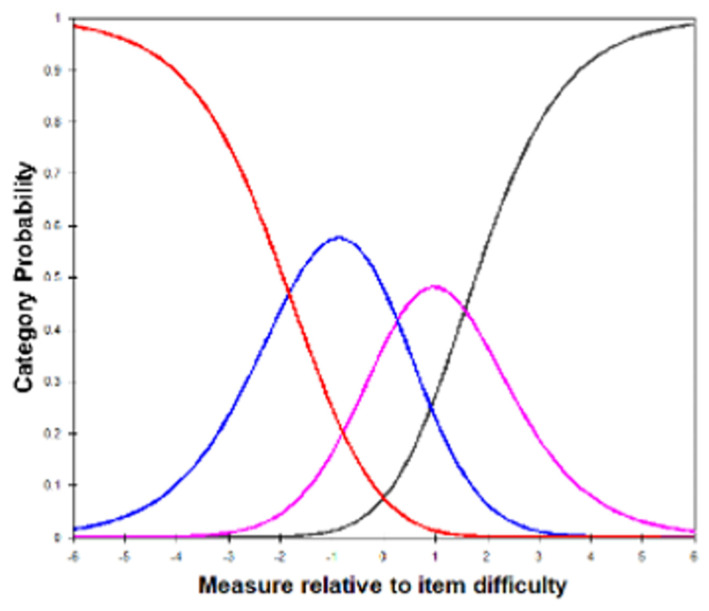
Category probability curve for responses to the metamorphopsia questionnaire (MeMoQ) in the patients with nAMD treated with Anti-VEGF on initial Rasch analysis. Each curve represents the probability of selection of a response category relative to the item’s difficulty. Category 1 (red) is more likely for “easier” items; then, category 2 (blue) is more likely to be selected for “moderately easy”, category 3 (pink) for “moderately difficult” and category 4 (black) for “difficult” items.

There were 1077 responses from patients with nAMD undergoing Anti-VEGF treatment. Initial visual inspection of category probability curves (Figure 2) demonstrated reasonable ordering of categories with adequate spacing of Andrich thresholds greater than 1.2 logits. However, person separation of the eight-item model was poor, achieving only a value of 1.47 with 68% reliability. This is supported by examination of the person-item map which demonstrated poor alignment of the item difficulty with the person ability with a difference in the means of −2.92 logits.

Furthermore, the fit statistics of the initial model widely ranged, with infit mean square residuals from 0.54 to 2.05, and outfit statistics from 0.54 to 2.2 (Table 3). The model is further undermined by a degree of multidimensionality with three items loading greater than 0.4 onto the first contrast which possessed a significant eigenvalue of 2.02. These items all assessed aspects of vision within the patient’s home environment (3. Do the curtain rails in your house appear distorted or tilted to you?; 4. Do the frames of windows or bookshelves appear distorted to you?; and 5. Do the lines of the tiles on your bathroom wall appear distorted to you?).

Iterative refinement of the MeMoQ for the 1077 responses for patients with nAMD was performed a total of four times until all items retained an infit mean square residual between 0.8 and 1.2. This results in a three-item questionnaire (3. Do the curtain rails in your house appear distorted or tilted to you?; 4. Do the frames of windows or bookshelves appear distorted to you?; and 5. Do the lines of the tiles on your bathroom wall appear distorted to you? Table S2; Figure S2), which aligned with those loading on the alternate construct in the original model.

The model improved person separation, approaching acceptable level at 1.98 with 80% reliability of categorising a person into the relevant stratum that reflected their level of ability. The model retained good category ordering with adequate spacing of category thresholds of more than 1.20 logits.

The three-item questionnaire demonstrated improved unidimensionality with an eigenvalue of 1.34 in the first contrast suggesting that there was no significant loading of any two of the three items into a different construct. This was supported by improved fitting of the infit mean square residuals with a maximum of 1.13; however, a less stringent lower value of 0.75 was accepted given the low number of retained items in the final questionnaire.

### 3.3. Subgroup Analysis of Patients Being Treated for DMO with Intravitreal Anti-VEGF

In this smaller subgroup of 281 responses the results were poorer than the entire population, with a person separation of 0.73 and a reliability of 0.35. Although there was good category ordering and spacing with >1.2 logits between Andrich thresholds, items were poorly fitting ranging from 0.58 to 1.89 for infit and 0.56 to 1.77 for outfit statistics (Table S3; Figure S3). The model also lacked unidimensionality with three items attaining a loading of >0.4 on the PCA with an eigenvalue of 2.5.

A single iteration of optimisation by removal of misfitting items was performed resulting in a three-item questionnaire (1. Do the lines of a crosswalk or the steps of an overpass appear distorted to you?; 3. Do the curtain rails in your house appear distorted or tilted to you?; and 6. Does the outline of your television set appear distorted or tilted to you? Table S4; Figure S4). This model, however, performed poorly with person separation of 0.18 and reliability of 0.03.

### 3.4. Subgroup Analysis of Patients Being Treated for Macular Oedema Associated with RVO

There were 282 individuals in the population being treated with Anti-VEGF for macular oedema associated with RVO. The eight-item MeMoQ achieved person separation that was inadequate at 1.32 with low reliability of 0.64, although with good category ordering and threshold spacing > 1.2 logits. Items were grossly misfitting, ranging from 0.51 to 2.08 for infit statistics and 0.44 to 2.42 for outfit statistics (Table S5; Figure S5).

Eliminating those items that are outside the infit statistic range from 0.8 to 1.2 resulted in a two-item questionnaire (1. Do the lines of a crosswalk or the steps of an overpass appear distorted to you?; 7. Does your face appear distorted to you in the mirror? Table S6; Figure S6). The performance of this questionnaire was worse than the eight-item MeMoQ with person separation of 0.95 and reliability of 0.47. As such, further statistics relating to this model were not useable.

## 4. Discussion

The current study provides evidence that in this large population of patients with macular disease the MeMoQ appears to perform inadequately to provide robust psychometric insight into a patient’s symptomatology.

Although item categories showed good ordering when inspected visually, this did not translate to a reliable measure across the entire questionnaire to provide detailed insight into a patient’s experience. This was primarily driven by grossly misfitting items in the entire population, suggesting both elements with less than expected variance and hence redundancy in those items, but also increased variance suggesting that the questionnaire’s items might not be targeting a similar construct. Thus, after removal of such items the MeMoQ was reduced to two items, which was inadequate to provide any reliable person separation and thus not provide any real-world insight into a patient’s symptomatic burden. This is underpinned by the fact that two items would be insufficient to capture the full construct of metamorphopsia, compromising content validity, and would not reflect sufficient clinical relevance. Therefore, it would be likely that such a brief questionnaire would not provide a true and reliable reflection of patient’s symptomatology of metamorphopsia or provide any additional usable information to clinicians when treating patients with macular diseases.

Another area for concern with the questionnaire was the considerable mistargeting of the questionnaire with a difference of 3.27 logits between item difficulty and person “ability”. This may reflect numerous aspects of the underlying assumptions of the original questionnaire which was developed in the context of patients with epiretinal membrane, macular hole or AMD [13]. Therefore, it may limit the applicability of the MeMoQ in the clinical setting where it has been used in macular diseases being treated with Anti-VEGF, an issue this publication aims to address. Furthermore, the possibility of Anti-VEGF treatment reducing symptom variability is a plausible explanation, but is contradicted as the various underlying pathologies from nAMD, CMO from RVO and DMO all appear to have varied and incomplete reduction in objectively measured metamorphopsia [16,17,18,19]. It is, thus, difficult to assess if the observed mistargeting of the MeMoQ reflects the effects of Anti-VEGF therapy and a mismatch between the instrument and the population or inherent instrument failure. That being said, the clinical applicability of MeMoQ in patients undergoing Anti-VEGF treatment seems flawed based on this mistargeting regardless of its causation.

Multidimensionality appeared to be another facet in which the MeMoQ was undermined in this population of patients treated with Anti-VEGF. It appeared that there might be a subdomain of clustering items that reflected the patient’s home environment (3. Do the curtain rails in your house appear distorted or tilted to you?; 4. Do the frames of windows or bookshelves appear distorted to you?; 5. Do the lines of the tiles on your bathroom wall appear distorted to you?). This may have undermined the overall questionnaires utility but none of these items functioned in a reliable manner with acceptable fit statistics. However, this is of interest for the development of future PROMs aiming to assess metamorphopsia as it may impact questionnaire design to avoid this or incorporate a relevant subdomain for patients, something that is not addressed in the widely used 25-item National Eye Institute Visual Functioning Questionnaire (NEI VFQ-25) [28].

When different disease subgroups were examined, the functionality of the MeMoQ did not improve despite iterative refinement of items within the questionnaire. This was true for patients with DMO, CMO associated with RVO and nAMD. Albeit, in the RVO and DMO groups there may be more clinical heterogeneity within the small sample size, and it is difficult to attribute the lack of psychometric robustness to this or the MeMoQ instrument itself. This all suggests that in the context of macular disease treated with Anti-VEGF the MeMoQ may not be appropriate for this particular population. Some factors that may contribute to this could be the translation of the original Japanese questionnaire to an English version, which has been the version widely utilised in Western populations [13,14,29]. Since this study population is predominantly rural in residency and first language English or Welsh, the specifics of the original questionnaire that provide psychometrically robust outcomes in Japan do not appear to be sustained. Although a lot of the questions reflect those typically targeted at individuals who suffer from metamorphopsia and are typical of questions suggested to practicing clinicians to discuss symptoms of visual distortion they do not function robustly as a single questionnaire. This is evident from the application of the questionnaire in the setting of nAMD, where participants reported a high frequency of such symptoms, with higher objective measures of metamorphopsia also correlating with worse vision-related quality of life as reported by the NEI VFQ-25 [30].

There is a paucity of evidence regarding the use of the MeMoQ, especially in the English language, where it has gained significant use in routine clinical practice [14,15]. Of studies that have utilised the questionnaire only the original published article assesses patients with AMD or other non-surgical causes of metamorphopsia, with one study conducted in French with no apparent validation of the translated instrument, which is also the case for a prior case of the questionnaire’s application in English [13,14,31]. In addition to this paucity of evidence it is worth noting that the questionnaire was originally validated in patients suffering from binocular or uniocular metamorphopsia, and the effects of uniocular disease and interocular suppression have not been fully explored in the context of MeMoQ [13,32].

Despite the MeMoQ not functioning, those suffering from metamorphopsia are an ever-expanding group with the ageing population and associated increasing prevalence of nAMD, DMO and CMO associated with RVO [22,33,34]. This is only likely to continue with increasing pressures on healthcare providers to treat these individuals with more patient-outcome focused care pathways [35]. As the therapeutic options for such a macular disease are expanding from the first-generation Anti-VEGF to subsequent biosimilars, it will also be important to assess patient outcomes to novel differing therapies that appear to offer similar outcomes in terms of the reductive visual acuity testing used in larger randomised control trials [2]. It is important to consider that, in this context, the current study does not appear to support the use of the MeMoQ in those undergoing treatment with Anti-VEGF for a range of macular diseases, and as such it would not appear productive for clinicians to administer the questionnaire in this particular setting.

In the wider context, there is increasing emphasis on creating instruments that demonstrate strong psychometric performance across diverse populations in ophthalmology [36]. Current evidence indicates that PROMs in eye care may lack robust validation for the specific populations in which they are used, highlighting the need for more rigorous population-specific development and testing [37]. Rasch analysis has become integral to instrument refinement for vision-related questionnaires, enabling assessment of item targeting, fit and category functioning, as well as guiding the removal of poorly performing items [23]. This is particularly relevant for vision-related questionnaires, in which symptoms like metamorphopsia may require sensitive measurement tools. Yet, the variability in available metamorphopsia assessment methods, e.g., Amsler grids, M-Charts, and preferential hyperacuity perimetry, each capturing different aspects of distortion with differing sensitivity, highlights the challenge in quantifying a subjective and heterogenous symptom [38].

Traditional Amsler grids provide quick, qualitative detection of distortion, but are highly subjective and confounded by fixation instability [39]. M-Charts quantify distortion magnitude more reliably, using a psychophysical estimate of perceived distortion [38,40], yet still assess metamorphopsia as a detection task rather than a functional symptom. Preferential hyperacuity perimetry measures vernier hyperacuity thresholds to detect subtle macular changes, functioning as a sensitive performance-based measure [41]. By contrast, the MeMoQ captures metamorphopsia conceptually as a patient-reported functional visual disturbance. Such conceptual mismatch, i.e., detection task vs. reporting of living experience, may help explain the limited psychometric targeting in this population.

Limitations of the current study include its retrospective analysis of a large cohort of heterogenous patients who have undergone a varying number of treatments with Anti-VEGF as well as responded to the questionnaire on multiple occasions or just once in a large number of cases. This has implications in terms of intra-user dependence of responses. This is of particular relevance when considering that all responses were treated as independent observations, even where they were repeated responses by individuals at different time points as this could lead to bias with overfitting and underestimation of the true variance due to effects of memory, learning and adaptation. However, it is worth noting the poor fitting of items in many of the scenarios discussed within this data set. In terms of Rasch analysis, differential item functioning was not considered in this study, as due to the poorly fitting articles it was of limited interpretability. In addition, it is worth noting this was distributed digitally to a predominantly more elderly population and issues of digital literacy may affect those able to respond to the questionnaire, potentially contributing to bias. Furthermore, patients may have received other intravitreal therapies at other time points, but this may not have been captured in the questionnaires’ data collection.

## 5. Conclusions

Overall, in this large population of English-speaking patients, the translated MeMoQ appears to function poorly and does not provide psychometrically robust insights into patient-reported outcome measures. Whether this be due to the translation or due to the specific content of questions is unclear. Further work to provide a reliable and robust PROM to report severity of symptoms for patients with macular disease experiencing significant distortion and metamorphopsia should be explored to provide better patient-centred care of this population. Crucial to ensuring the PROM reflects real-world experiences for patients and is clinically relevant would be further assessment of the content validity. This should include assessment of terminology and concepts explored within the questions of the MeMoQ, engaging with all key stakeholders including patients with lived experience as well as retina specialists with specialist knowledge of macular pathology and emerging therapeutic options for such diseases.

## Figures and Tables

**Table 1:** The abbreviated metamorphopsia questionnaire (MeMoQ). This summarises the 8-item questionnaire adapted from the original 10-item MeMoQ with elimination of the one item that was previously demonstrated to be misfitting and one item deemed inappropriate for the European population.

Item Number	Items
1	Do the lines of a crosswalk or the steps of an overpass appear distorted to you?
2	Do telephone poles or trees appear tilted to you?
3	Do the curtain rails in your house appear distorted or tilted to you?
4	Do the frames of windows or bookshelves appear distorted to you?
5	Do the lines of the tiles on your bathroom wall appear distorted to you?
6	Does the outline of your television set appear distorted or tilted to you?
7	Does your face appear distorted to you in the mirror?
8	When reading a book, newspaper or display on a computer screen, do the lines of words appear distorted to you?

**Table 2:** Item measure and Rasch fit statistics for the MeMoQ in the entire population of patients being treated with intravitreal Anti-VEGF in HDUHB.

Item Number	Items	Item Measure (Logits)	Infit (MNSQ)	Outfit (MNSQ)
1	Do the lines of a crosswalk or the steps of an overpass appear distorted to you?	−0.71	1.11	1.17
2	Do telephone poles or trees appear tilted to you?	0.28	0.9	0.87
3	Do the curtain rails in your house appear distorted or tilted to you?	0.11	0.57	0.55
4	Do the frames of windows or bookshelves appear distorted to you?	−0.09	0.55	0.54
5	Do the lines of the tiles on your bathroom wall appear distorted to you?	0.15	0.6	0.58
6	Does the outline of your television set appear distorted or tilted to you?	0.34	0.69	0.61
7	Does your face appear distorted to you in the mirror?	1	1.42	1.21
8	When reading a book, newspaper or display on a computer screen, do the lines of words appear distorted to you?	−1.07	2.19	2.4

**Table 3:** Item measure and rasch fit statistics for the MeMoQ in patients being treated for nAMD.

Item Number	Items	Item Measure (Logits)	Infit (MNSQ)	Outfit (MNSQ)
1	Do the lines of a crosswalk or the steps of an overpass appear distorted to you?	−0.7	1.12	1.19
2	Do telephone poles or trees appear tilted to you?	0.17	0.91	0.9
3	Do the curtain rails in your house appear distorted or tilted to you?	−0.02	0.54	0.54
4	Do the frames of windows or bookshelves appear distorted to you?	−0.13	0.56	0.54
5	Do the lines of the tiles on your bathroom wall appear distorted to you?	0.07	0.64	0.65
6	Does the outline of your television set appear distorted or tilted to you?	0.31	0.73	0.67
7	Does your face appear distorted to you in the mirror?	1.14	1.59	1.37
8	When reading a book, newspaper or display on a computer screen, do the lines of words appear distorted to you?	−0.85	2.05	2.2

## Data Availability

The datasets used and/or analysed during the current study are available from the corresponding author on reasonable request.

## Supplementary Materials

The following supporting information can be downloaded at https://www.mdpi.com/article/10.3390/jcm15082867/s1.

## Abbreviations

The following abbreviations are used in this manuscript:

Anti-VEGF	Anti-Vascular Endothelial Growth Factor
CMO	Cystoid Macular Oedema
DMO	Diabetic Macular Oedema
HDUHB	Hywel Dda University Health Board
MeMoQ	Metamorphopsia Questionnaire
nAMD	Neovascular Age-Related Macular Degeneration
PCA	Principal Component Analysis
PROM	Patient-Reported Outcome Measure
RVO	Retinal Vein Occlusion
