# Supplementary material for: Psychometric Assessment of the Metamorphopsia Questionnaire in Patients with Macular Diseases Receiving Anti-Vascular Endothelial Growth Factor Treatment
Source: J Clin Med. 2026 Apr 9;15(8):2867. doi: 10.3390/jcm15082867 (PMC13116337; doi:10.3390/jcm15082867)
Supplement: Supplementary file 1 [file jcm-15-02867-s001.zip › jcm-4151462-supplementary.pdf]

Table S1 Item Measure And Rasch Fit Statistics For The MeMoQ In Entire Population Patients Being Treated with Intravitreal Anti-VEGF in HDUHB after Iterative Refinement

Item Number	Items	Item Measure (Logits)	Infit (MNSQ)	Outfit (MNSQ)
1	Do the lines of a crosswalk or the steps of an overpass appear distorted to you?	-1.05	1.03	1.05
2	Do telephone poles or trees appear tilted to you?	1.05	0.93	0.90

Table S2 Item Measure And Rasch Fit Statistics For The MeMoQ In Patients Being Treated For nAMD after Iterative Refinement

Item Number	Items	Item Measure (Logits)	Infit (MNSQ)	Outfit (MNSQ)
3	Do the curtain rails in your house appear distorted or tilted to you?	0.02	1.07	0.95
4	Do the frames of windows or bookshelves appear distorted to you?	-0.34	0.75	0.65
5	Do the lines of the tiles on your bathroom wall appear distorted to you?	0.32	1.13	0.97

Table S3 Initial Item Measure And Rasch Fit Statistics For The MeMoQ In Patients Being Treated for DMO with Intravitreal Anti-VEGF in HDUHB

Item Number	Items	Item Measure (Logits)	Infit (MNSQ)	Outfit (MNSQ)
1	Do the lines of a crosswalk or the steps of an overpass appear distorted to you?	-0.67	1.04	1.02
2	Do telephone poles or trees appear tilted to you?	0.36	0.71	0.87
3	Do the curtain rails in your house appear distorted or tilted to you?	0.61	0.91	0.77
4	Do the frames of windows or bookshelves appear distorted to you?	0.1	0.58	0.56
5	Do the lines of the tiles on your bathroom wall appear distorted to you?	0.32	0.63	0.61
6	Does the outline of your television set appear distorted or tilted to you?	0.4	0.82	0.6
7	Does your face appear distorted to you in the mirror?	0.61	1.34	1.03
8	When reading a book, newspaper or display on a computer screen, do the lines of words appear distorted to you?	-1.73	1.89	1.77

Table S4 Item Measure And Rasch Fit Statistics For The MeMoQ In Patients Being Treated For DMO after Iterative Refinement

Item Number	Items	Item Measure (Logits)	Infit (MNSQ)	Outfit (MNSQ)
1	Do the lines of a crosswalk or the steps of an overpass appear distorted to you?	-1.1	1.16	1.12
3	Do the curtain rails in your house appear distorted or tilted to you?	0.69	0.9	0.84
6	Does the outline of your television set appear distorted or tilted to you?	0.41	0.94	0.91

Table S5 Initial Item Measure And Rasch Fit Statistics For The MeMoQ In Patients Being Treated for RVO with Intravitreal Anti-VEGF in HDUHB

Item Number	Items	Item Measure (Logits)	Infit (MNSQ)	Outfit (MNSQ)
1	Do the lines of a crosswalk or the steps of an overpass appear distorted to you?	-0.42	0.86	0.85
2	Do telephone poles or trees appear tilted to you?	0.82	1.73	1.81
3	Do the curtain rails in your house appear distorted or tilted to you?	0.19	0.57	0.48
4	Do the frames of windows or bookshelves appear distorted to you?	-0.17	0.55	0.48
5	Do the lines of the tiles on your bathroom wall appear distorted to you?	0.29	0.51	0.44
6	Does the outline of your television set appear distorted or tilted to you?	0.22	0.58	0.51
7	Does your face appear distorted to you in the mirror?	0.82	1.05	0.99
8	When reading a book, newspaper or display on a computer screen, do the lines of words appear distorted to you?	-1.74	2.08	2.42

Table S6 Item Measure And Rasch Fit Statistics For The MeMoQ In Patients Being Treated for RVO with Intravitreal Anti-VEGF in HDUHB after Iterative Refinement

Item Number	Items	Item Measure (Logits)	Infit (MNSQ)	Outfit (MNSQ)
1	Do the lines of a crosswalk or the steps of an overpass appear distorted to you?	-1.14	0.97	0.89
7	Does your face appear distorted to you in the mirror?	1.14	1.02	1.00

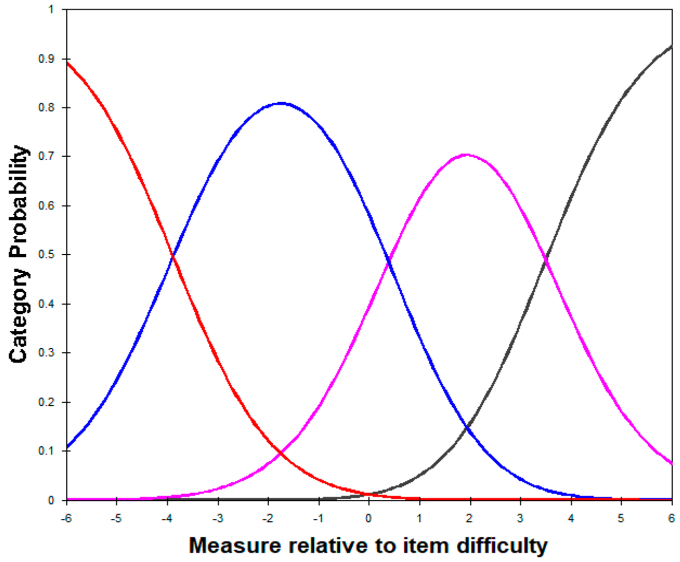

Figure S1 Category-Probability curve for responses to the 2-item Metamorphopsia Questionnaire (MeMoQ) in the entire population after iterative Rasch analysis. Each curve represents the probability of selection of a response category relative to the item's difficulty. Category 1 (red) is more likely for 'easier' items, then category 2 (blue) is more likely to be selected for 'moderately easy', category 3 (pink) for 'moderately difficult' and category 4 (black) for 'difficult' items.

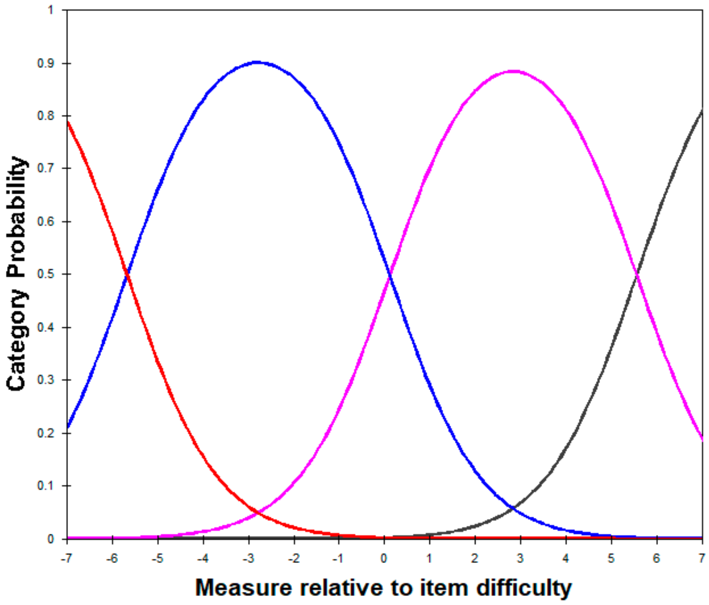

Figure S2 Category-Probability curve for responses to the 3-item Metamorphopsia Questionnaire (MeMoQ) in patients with age-related macular degeneration (nAMD) treated with Anti-VEGF after iterative Rasch analysis. Each curve represents the probability of selection of a response category relative to the item's difficulty. Category 1 (red) is more likely for 'easier' items, then category 2 (blue) is more likely to be selected for 'moderately easy', category 3 (pink) for 'moderately difficult' and category 4 (black) for 'difficult' items.

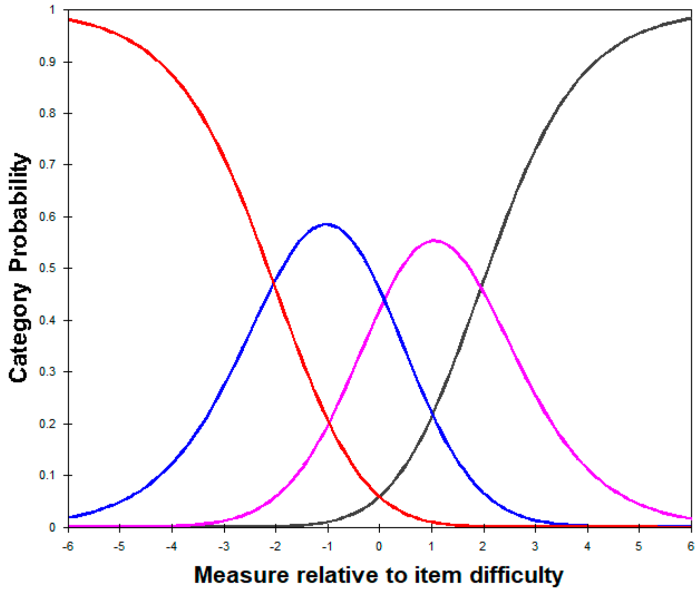

Figure S3 Category-Probability curve for responses to the Metamorphopsia Questionnaire (MeMoQ) in patients with diabetic macular oedema (DMO) treated with Anti-VEGF on initial Rasch analysis. Each curve represents the probability of selection of a response category relative to the item's difficulty. Category 1 (red) is more likely for 'easier' items, then category 2 (blue) is more likely to be selected for 'moderately easy', category 3 (pink) for 'moderately difficult' and category 4 (black) for 'difficult' items.

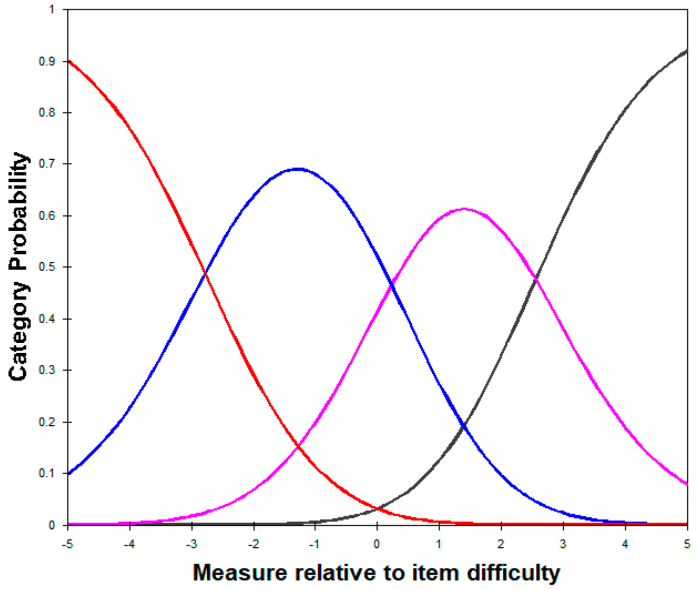

Figure S4 Category-Probability curve for responses to the 3-item Metamorphopsia Questionnaire (MeMoQ) in patients with diabetic macular oedema (DMO) treated with Anti-VEGF after iterative Rasch analysis. Each curve represents the probability of selection of a response category relative to the item's difficulty. Category 1 (red) is more likely for 'easier' items, then category 2 (blue) is more likely to be selected for 'moderately easy', category 3 (pink) for 'moderately difficult' and category 4 (black) for 'difficult' items.

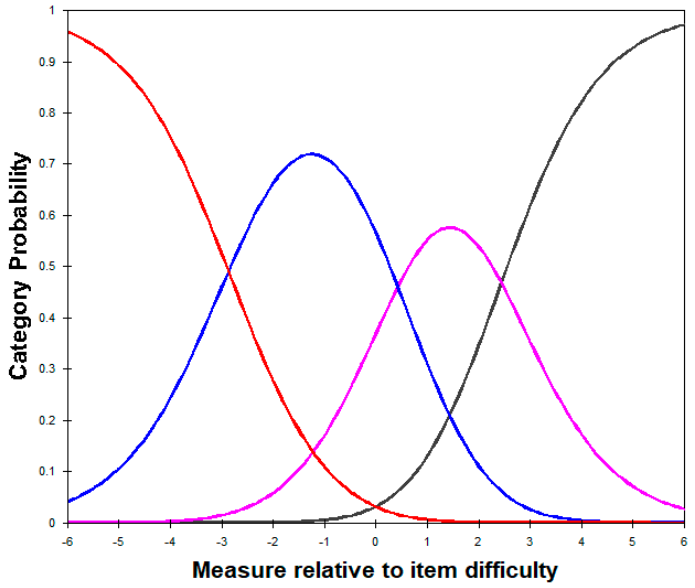

Figure S5 Category-Probability curve for responses to the Metamorphopsia Questionnaire (MeMoQ) in patients with cystoid macular oedema associated with retinal vein occlusion (RVO) treated with Anti-VEGF on initial Rasch analysis. Each curve represents the probability of selection of a response category relative to the item's difficulty. Category 1 (red) is more likely for 'easier' items, then category 2 (blue) is more likely to be selected for 'moderately easy', category 3 (pink) for 'moderately difficult' and category 4 (black) for 'difficult' items.

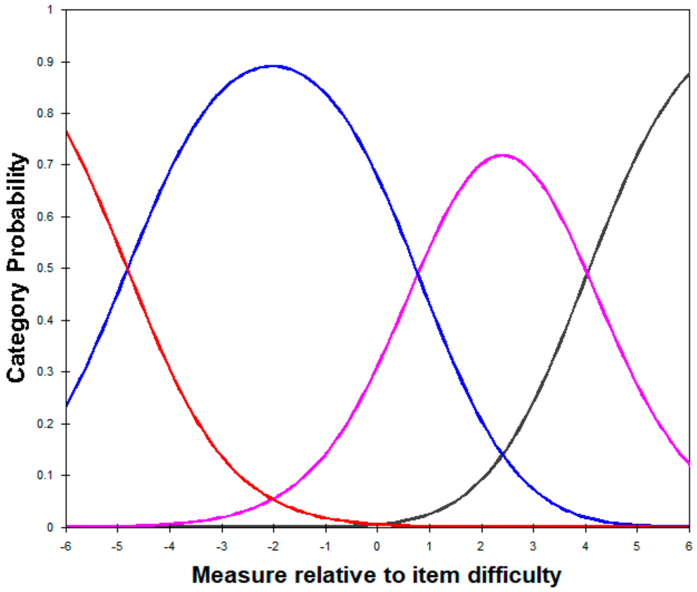

Figure S6 Category-Probability curve for responses to the 2-item Metamorphopsia Questionnaire (MeMoQ) in patients with cystoid macular oedema associated with retinal vein occlusion (RVO) treated with Anti-VEGF after iterative Rasch analysis. Each curve represents the probability of selection of a response category relative to the item's difficulty. Category 1 (red) is more likely for 'easier' items, then category 2 (blue) is more likely to be selected for 'moderately easy', category 3 (pink) for 'moderately difficult' and category 4 (black) for 'difficult' items.
